# Estimation of the epidemiological burden of human papillomavirus-related cancers and non-malignant diseases in men in Europe: a review

**DOI:** 10.1186/1471-2407-12-30

**Published:** 2012-01-20

**Authors:** Susanne Hartwig, Stina Syrjänen, Géraldine Dominiak-Felden, Maria Brotons, Xavier Castellsagué

**Affiliations:** 1 Department of Epidemiology, Sanofi Pasteur MSD, Lyon, France; 2 Department of Oral Radiology and Pathology, Faculty of Medicine, Institute of Dentistry and MediCity Research Laboratory, Turku, Finland; 3 Cancer Epidemiology Research Program, L'Hospitalet de Llobregat, Institut Català d'Oncologia (ICO)-IDIBELL, Catalonia, Spain; 4 CIBER en Epidemiología y Salud Pública (CIBERESP), Barcelona, Spain

**Keywords:** HPV, Epidemiology, Cancer, Genital warts, Condylomata acuminata, Men, Europe

## Abstract

**Background:**

The role of human papillomavirus (HPV) in malignant and non-malignant genital diseases in women is well known and the corresponding epidemiological burden has been widely described. However, less is known about the role of HPV in anal, penile and head and neck cancer, and the burden of malignant and non-malignant HPV-related diseases in men. The objective of this review is to estimate the epidemiological burden of HPV-related cancers and non-malignant diseases in men in Europe.

**Methods:**

The annual number of new HPV-related cancers in men in Europe was estimated using Eurostat population data and applying cancer incidence rates published by the International Agency for Research on Cancer. The number of cancer cases attributable to HPV, and specifically to HPV16/18, was calculated based on the most relevant prevalence estimates. The annual number of new cases of genital warts was calculated from the most robust European studies; and latest HPV6/11 prevalence estimates were then applied. A literature review was also performed to retrieve exhaustive data on HPV infection at all anatomical sites under study, as well as incidence and prevalence of external genital warts, recurrent respiratory papillomatosis and HPV-related cancer trends in men in Europe.

**Results:**

A total of 72, 694 new cancer cases at HPV-related anatomical sites were estimated to occur each year in men in Europe. 17,403 of these cancer cases could be attributable to HPV, with 15,497 of them specifically attributable to HPV16/18. In addition, between 286,682 and 325,722 new cases of genital warts attributable to HPV6/11were estimated to occur annually in men in Europe.

**Conclusions:**

The overall estimated epidemiological burden of HPV-related cancers and non-malignant diseases is high in men in Europe. Approximately 30% of all new cancer cases attributable to HPV16/18 that occur yearly in Europe were estimated to occur in men. As in women, the vast majority of HPV-positive cancer in men is related to HPV16/18, while almost all HPV-related non-malignant diseases are due to HPV6/11. A substantial number of these malignant and non-malignant diseases may potentially be prevented by quadrivalent HPV vaccination.

## Background

Human papillomavirus (HPV) has been established as a necessary cause of cervical cancer. Around 50% of women become infected with HPV by age 20-30 years [1] and persistent infection can lead to the development of malignant and non-malignant diseases. Indeed, the role of HPV in cervical cancer, in premalignant lesions of the cervix, vulva and vagina, and in external genital warts is well known, and the corresponding epidemiological burden in women has been widely described [2-6]. However, less is known about the natural history of HPV infection in men and the role of HPV in penile, anal and head and neck cancers.

As early as 1983, it was suggested that a subset of cancers of the oral cavity and larynx may be caused by HPV [7]. The juxtaposition between the squamous cell epithelium and the lymphatic tissue in the oropharynx, as well as the transformation zone of the anal canal, share similarities with the transformation zone of the cervix, and might therefore be areas that are highly susceptible to HPV infection.

In 2007 the International Agency for Research on Cancer (IARC) concluded that there was "sufficient" evidence to support the carcinogenicity of HPV in the penis, anus, oral cavity, oropharynx and tonsils, and "limited" evidence to support the carcinogenicity of HPV in the larynx [8]. This statement was confirmed by an IARC Monograph Working Group in 2011 [9].

In addition to cancer, HPV is responsible for some non-malignant diseases. Indeed, the great majority of genital warts, recurrent respiratory papillomatosis and oral papillomas are attributable to HPV, mostly HPV6/11 [10-12].

All HPV-induced cancers and non-malignant diseases are necessarily preceded by HPV infection. However, little is known about the prevalence of HPV infection and related diseases in men at different anatomical sites. Therefore the purpose of this review is to estimate the epidemiological burden of HPV-related cancers and non-malignant diseases, as well as the prevalence of HPV infection, in men in Europe.

## Methods

### Literature review

A literature review was performed to retrieve exhaustive European data on trends of HPV-related cancers, incidence and prevalence of genital warts, recurrent respiratory papillomatosis and HPV infection. Searches were conducted on MEDLINE and included articles published from January 1990 through November 2010.

Articles containing data on the trends of HPV-related cancers were obtained by combining the search terms "HPV", "human papillomavirus", "cancers" and "trends". Those with data on the prevalence and incidence of genital warts and recurrent respiratory papillomatosis were obtained by using different combinations of the following search terms "genital warts", "anogenital warts", "external warts", "condyloma/condylomata acuminata", "incidence" and "prevalence"; and "papillomatosis", "incidence" and "prevalence", respectively. Finally, articles containing data on the incidence and prevalence of HPV infections were obtained by combining the search terms "HPV", "human papillomavirus", "infection", "oral cavity", "oropharynx", "larynx", "anus", "penis", "men" and "males".

Only articles referring to the populations of the 26 European countries included in this review were considered. Articles identified from the reference lists of retrieved publications were also included.

### Estimation of the annual number of new cancer cases

For the purposes of this review, cancers occurring at three anatomical sites were considered HPV-related, as HPV infection may play a role in their development: a subset of head and neck cancers (International Classification of Diseases 10^th ^Revision (ICD-10) codes C01-C02, C03-C06, C09, C10, C12, C13, C14 and C32, (Table 1)), anal cancer (C21) and penile cancer (C60). All epidemiological cancer data described in this review refer exclusively to these selected cancers and cancer sites, which are hereafter referred to as "HPV-related".

**Table 1:** List of included head and neck sites and subsites

Site description	ICD-10 Code
**Oral cavity**	

Tongue (incl. base of the tongue, other parts of the tongue)	C01-C02

Mouth (incl. gum, floor of the mouth, palate)	C03-C06

**Pharynx**	

Tonsil	C09

Oropharynx	C10

Piriform sinus	C12

Hypopharynx	C13

Pharynx unspecified (incl. Waldeyer's ring, overlapping lesion of lip, oral cavity and pharynx)	C14

**Larynx**	C32

To estimate the mean annual number of new HPV-related cancer cases in men in Europe, sex- and age- specific annual cancer incidence rates by country, extracted from Volume IX of the Cancer Incidence in Five Continents (CI5) database, were applied to the respective Eurostat country male population estimates [13]. The age-specific estimated numbers of new male cancer cases were summed up to obtain the overall number of expected cases in 2008 for each selected country:

$$\overset{85+}{\underset{0}{\sum }}nb\;of\;new\;cases\;2008=\frac{AIR*\left(per\;100,000\right)\times population**\left(2008\right)}{100,000}$$

*age- and sex-specific annual incidence rate

**age- and sex-specific population

The expected numbers of new cancer cases of all selected countries were then summed up to estimate the overall European Burden.

The number of cancer cases that may have been attributable to HPV and specifically to HPV16/18 was then evaluated by applying cancer-specific HPV prevalence and HPV16/18 prevalence estimates, respectively, extracted from the most relevant published data. When available, European data were used. When European data were not available, worldwide data were used.

For comparison purposes, the same methods were also used to estimate the epidemiological burden of HPV-related cancers in women in Europe. HPV-related cancer sites in women are the cervix uteri (ICD-10 code C53), vagina (C52), vulva (C51), anus (C21) and a subset of head and neck cancers (C01-C02, C03-C06, C09, C10, C12, C13, C14 and C32).

The CI5 database, available on the IARC website [14], contains worldwide data on cancer incidence rates by ICD-10 codes, obtained from cancer registries that meet the IARC's quality criteria [15]. The registries are either regional or national, depending on the country. The data included in Volume IX were collected between 1998 and 2002.

Only cancer registries that meet the IARC's quality criteria, i.e., that have reliable national or regional cancer registry data, are included in the CI5 database. We selected all European Union countries. Four of them (Greece, Hungary, Luxemburg, and Romania) were not included in the CI5 database as they did not have reliable registry data. Three European countries (Iceland, Norway and Switzerland) that do not belong to the European Union were also selected as they are located in the same geographical area and share similarities in terms of way of life. Hence a total of 26 European countries were included in this analysis.

Information in the CI5 database originated from national cancer registries for Austria, Bulgaria, Cyprus, the Czech Republic, Denmark, Estonia, Finland, Iceland, Ireland, Latvia, Lithuania, Malta, the Netherlands, Norway, Slovenia, Slovakia and Sweden. Regional cancer registry data were available for Belgium, France, Germany, Italy, Poland, Portugal, Spain, Switzerland and the United Kingdom.

To ensure that national populations were adequately represented in countries where only regional cancer registries exist, the geographical coverage and distribution of these registries was assessed.

### Estimation of the annual number of new cases of genital warts

To estimate the annual number of incident cases of genital warts, two European publications which provided, based on their design, the most robust incidence data for Europe, were selected [16,17]: both are retrospective cohort studies carried out in databases, including very large samples of routinely collected data. We extrapolated the value from each publication to all 26 European countries to provide a range of estimated new cases of genital warts that are expected to occur yearly in men in Europe. The German incidence data [17] were used to estimate the lower value, and the data from the United Kingdom [16] the upper value. The estimated prevalence of HPV6/11 in genital warts was then applied based on the only two European publications identified on the subject [10,12].

For comparison purposes the same method was used to estimate the epidemiological burden of HPV-related genital warts in women in Europe.

## Results

### HPV-related cancers

#### Head and neck cancers

**Figure 1:**
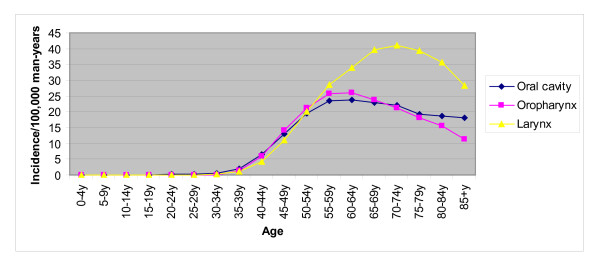
**Incidence of head and neck cancers, irrespective of HPV status, in men in Europe by age group**.

Head and neck cancers generally first occur between 30 and 35 years of age among men in Europe. The incidence then steadily increases with a peak in the sixth decade of life for oropharyngeal cancer and cancer of the oral cavity, and in the seventh decade of life for laryngeal cancer (Figure 1).

**Figure 2:**
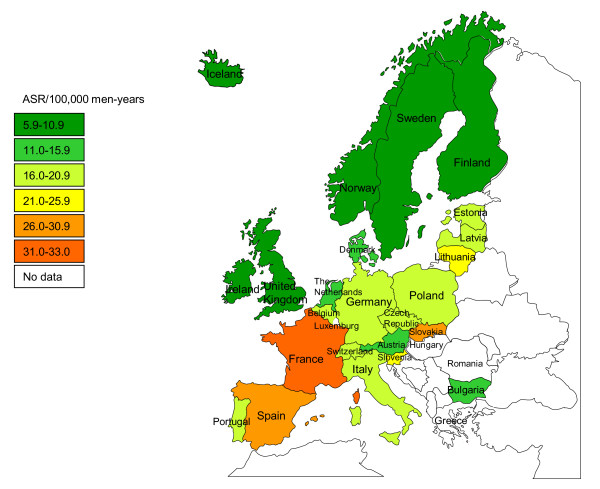
**Age-standardised incidence rate (ASR) of a subset of head and neck cancers irrespective of human papillomavirus status**.

The overall age-standardised incidence rates of the subset of head and neck cancers included in this review, irrespective of HPV status, ranged from 5.6 (in Cyprus) to 33.0 (in France) per 100,000 man-years in Europe (CI5 Volume IX) (Figure 2). They were characterised by a strong incidence gradient, with rates increasing from 5.6 to 8.2 per 100,000 man-years in Cyprus and the Nordic countries (Sweden, Finland, Norway and Iceland) up to 27.0-33.0 per 100,000 man-years in Spain, the Slovak Republic and France.

Among the included subset of head and neck cancers, laryngeal cancer had the highest incidence in Europe (age-standardised incidence rates ranging from 2.0 per 100,000 man-years in Sweden to 13.3 per 100,000 man-years in Spain), higher than that of cancer of the oral cavity (1.8 per 100,000 man-years in Cyprus to 10.7 per 100,000 man-years in France) and oropharynx (0.6 per 100,000 man-years in Cyprus to 14.1 per 100,000 man-years in France).

**Figure 3:**
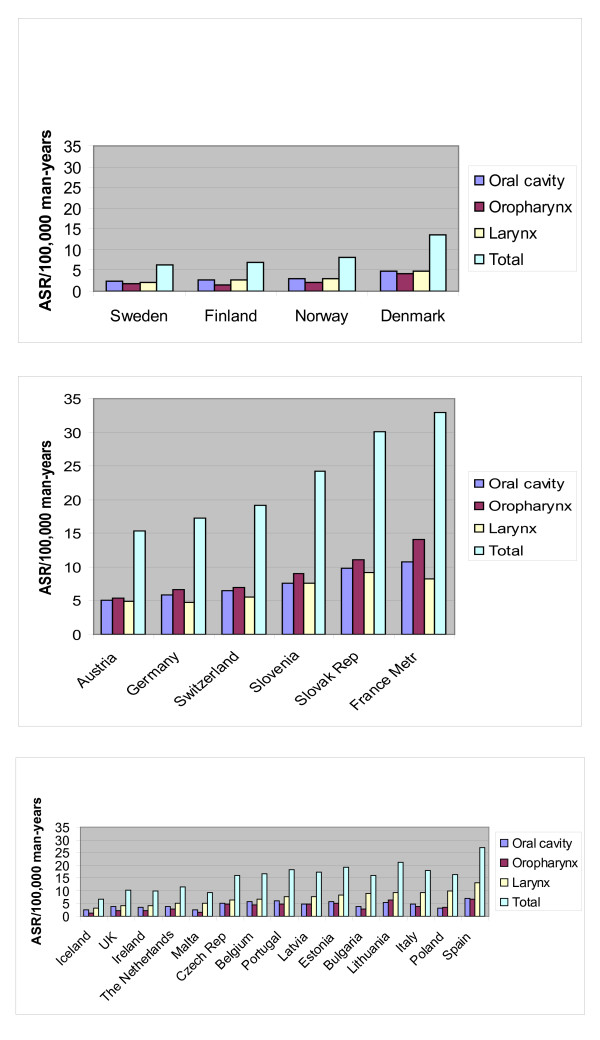
**Distribution of head and neck cancers by anatomical site in men in Europe**. a. Distribution of head and neck cancers by anatomical site in men in countries where cancer of the oral cavity is predominant. b. Distribution of head and neck cancers by anatomical site in countries where oropharyngeal cancer is predominant. c. Distribution of head and neck cancers by anatomical site in countries where laryngeal cancer is predominant. *ASR *age standardised incidence rate.

Moreover, the incidence of cancer at different head and neck sites did vary by geographical region. For example, cancer of the oral cavity was predominant in the Nordic countries, while oropharyngeal cancer was most common in Western and Central European countries (France, Germany, Switzerland, Austria, Slovenia and Slovakia). In the remaining European countries, laryngeal cancer was the most common, with incidence rates representing up to 50% of head and neck cancers in several countries (Bulgaria, Poland, Italy, Spain, Malta) (Figure 3a-c).

Within the included subset of head and neck cancers, a total of 67,354 (bounds: 63,443-71,292) new cases were estimated to occur in men in Europe every year (cancer of the oral cavity: 20,133 cases, oropharyngeal cancer: 20,433 cases, laryngeal cancer: 26,788 cases). Assuming an HPV prevalence of 16.0% for cancer of the oral cavity, 28.2% for oropharyngeal cancer (except hypopharyngeal cancer where HPV prevalence is the same as for laryngeal cancer) and 21.3% for laryngeal cancer [18], a total of 14,098 (bounds: 11,455-17,077) of these cases would actually be attributable to HPV (3,221 cancers of the oral cavity, 5,171 oropharyngeal cancers and 5,706 laryngeal cancers) (Table 2).

**Table 2:** Expected annual number of new human papillomavirus (HPV)-related cancer cases in men in Europe^a^

Cancer sites (ICD 10 code)	Expected number of new cancer cases, irrespective of HPV status (bounds)	HPV prevalence by site (%) (95%CI)	Expected number of new cancer cases attributable to HPV (bounds)	Prevalence of HPV 16/18 in HPV-positive cancers (%)	Expected number of new cancer cases attributable to HPV16/18
**Head and neck** Oral cavity^b^ Oropharynx^c^ Larynx (C32)	**67,354** (63,443-71,292) **20,133** (18,768-21,514) **20,433** (19,651-21,776) **26,788** (25,210-28,382)	**16.0** (13.4-18.8) **28.2^d^** (24.4-32.2) **21.3** (18.5-24.3) [18]	**14,098** (11,455-17,077) **3,221** (2,515-4,045) **5,171** (5030-5311) **5,706** (4,664-6,897)	**68.2/34.1** **86.7/2.8** **69.2/17.0**	**12,707** **3,221** **4,567** **4,919**

**Anus **(C21)	**2,162** (1,722-2,620)	**84.2** (81.5-86.9) [19]	**1,821** (1,403-2,277)	**87.1/6.2**	**1,699**

**Penis **(C60)	**3,178** (2,623-3,751)	**46.7** (42.0-51.3) [20]	**1,484** (1,102-1925)	**60.2/13.4**	**1,091**

**Total**	**72,694** (67,957-77,470)		**17,403** (14,049-21,161)		**15,497**

*ICD *International Classification of Diseases, *CI *confidence interval

^a ^26 European countries

^b ^Includes tongue (C01-02), mouth (C03-06), and also includes base of the tongue, which actually belongs to the oropharynx

^c ^Includes tonsil (C09), other oropharynx (C10), hypopharynx (C12-13) and pharynx unspecified (C14)

^d ^Except hypopharynx (C12-13), where HPV prevalence is the same as in laryngeal cancer (C32)

HPV16/18 are the predominant types in HPV-positive head and neck cancers. They are thought to be present in 68.2% and 34.1%, respectively, of HPV-positive cancers of the oral cavity, 86.7% and 2.8% of HPV-positive oropharyngeal cancers and 69.2% and 17.0% of HPV-positive laryngeal cancers [18]. Accordingly, a total of 12,707 new head and neck cancer cases were expected to be attributable to HPV16/18 each year in men in Europe (3,221 cancers of the oral cavity, 4,567 oropharyngeal cancers and 4,919 laryngeal cancers) (Table 2).

Three European studies reported an increasing incidence trend in the included subset of head and neck cancers in men. In the United Kingdom, the incidence of squamous cell carcinoma of the oral cavity and oropharynx in men rose by 51% between 1989 and 2006 [21]. In Sweden, an increase of 2.6% in the incidence of tonsillar cancer in men was observed in each year between 1960 and 2003 [22,23].

#### Anal cancer

**Figure 4:**
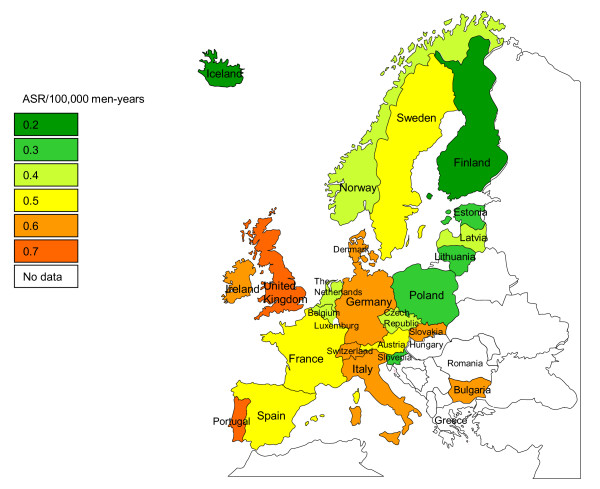
**Age-standardised incidence rate (ASR) of anal cancer irrespective of human papillomavirus status in men in Europe**.

Anal cancer generally first occurs between 30 and 35 years of age among men in Europe. The incidence then steadily increases with age in all European countries (data not shown). Age-standardised rates of anal cancer ranged from 0.2 (in Cyprus, Finland and Iceland) to 0.7 per 100,000 man-years (in the United Kingdom) (CI5 Volume IX) (Figure 4).

A total of 2,162 (bounds: 1,722-2,620) new anal cancer cases were estimated to occur each year in men in Europe, irrespective of HPV status. Given the overall HPV prevalence of 84.2% (95%CI: 81.5%-86.9%) [19] in anal cancer, 1,821 (bounds: 1,403-2,277) cases in men in Europe were estimated to be attributable to HPV. The prevalence of HPV16/18 in HPV positive anal cancer cases has been estimated at 87.1% and 6.2% respectively [19]. After applying this prevalence, 1,699 of cases were estimated to be attributable to HPV16/18 (Table 2).

Several studies have reported an increasing trend in the incidence of anal cancer in men in Europe over time. In Southeast England the age-standardised incidence rates in men increased from 0.79 per 100,000 man-years in 1960-1964 to 1.06 in 2000-2004. The Scottish Cancer Registry recorded an increase in the age-standardised incidence rates of anal squamous cell carcinoma in men, from 0.14 per 100,000 man-years in the late 1970s to 0.37 in the late 1990s, with a peak of 0.44 in 1993-1997 [24]. Similar trends have been seen in other European countries: in Denmark, the annual incidence rates in men rose from 0.25 per 100,000 man-years in 1958-1962 to 0.38 in 1983-1987 according to Frisch et al. [25] and the age standardised incidence rate of anal cancer in men increased from 0.45 per 100,000 man-years in 1978-1982 to 0.80 in 2003-2008 according to Nielsen et al. [26]. In Sweden, Goldman et al. [27] recorded an annual increase of 4.6% in incidence rates among men, from 0.52 per 100,000 man-years in 1970-1974 to 0.84 in 1980-1984. Increasing trends have also been reported from the Netherlands [28].

#### Penile cancer

**Figure 5:**
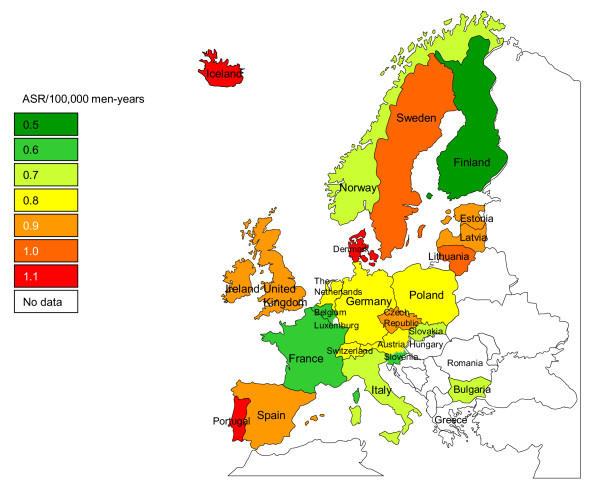
**Age-standardised incidence rate (ASR) of penile cancer irrespective of human papillomavirus status in men in Europe**.

In Europe, penile cancer tends to be diagnosed at age 30-35 years onwards, with an incidence peak in the seventh decade of life (data not shown). Age-standardised rates of penile cancer in men in Europe, irrespective of HPV status, ranged between 0.5 (in Finland) and 1.1 per 100,000 man-years (in Denmark, Portugal and Iceland) (CI5 Vol. IX) with substantial geographical variations (Figure 5).

A total of 3,178 (bounds: 2,623-3,751) new penile cancer cases were estimated to occur annually in Europe. Assuming an overall HPV prevalence of 46.7% (95%CI: 42.0%-51.3%) and a specific HPV16/18 prevalence of 60.2% and 13.4% respectively among HPV-positive cancers [20], 1,484 (bounds: 1,102-1,925) penile cancer cases were estimated to be attributable to HPV, with 1,091 of those specifically attributable to HPV16/18 (Table 2).

Increasing trends have been reported in the Netherlands, where one study noted a significant increase in the 3-year moving average incidence rate of penile squamous cell carcinoma between 1989 and 2006, with an estimated annual percentage of change of 1.3% [29].

### HPV-related non-malignant diseases

#### Genital warts

Genital warts represent a non-malignant disease affecting anogenital sites. In men genital warts may occur on the penis, anus, urethra, perianal skin and scrotum. Most reports in the scientific literature did not indicate the exact localisation of warts, and instead combined all localisations into one group.

##### Prevalence

Three studies provided data on the prevalence of genital warts in the general male population in Europe [30-32]. The lowest prevalence was reported in Spain (0.2%) [30], while the highest numbers were recorded in a cohort of 432 Finnish conscripts (5.6%) [31] (Table 3).

**Table 3:** Studies reporting on the incidence and prevalence of genital warts in general male populations in Europe^a^

Author, year, country	Population	Sample size	Design	Incidence/100,000	95%CI	Prevalence (%)	95%CI
Castellsagué, 2010 Spain [30]	General		Retrospective (medical records)	136		0.2	

Simms, 1997 UK [33]	GUM clinic attendees		Prospective	First occurence + recurrent cases 65 (1971) 319 (1994)		ND	

Cassell, 2006 UK [34]	General practice + GUM clinic attendees		Retrospective cohort	First occurence + recurrent cases 307.6	(305.5-309.8)	ND	

Kraut, 2010 Germany [17]		> 14,000,000	Retrospective cohort	144.57 (2005) 147.95 (2006)	(141.10-148.09) (144.48-151.48)	ND	

Fenton, 2001 UK [35]	General	4,500	Survey	Cumulative incidence (self reported) 360	(310-420)	ND	

Persson, 1996 Sweden [36]	General	32,774	Prospective	294	(207-372)	ND	

Hippeläinen, 1993 Finland [31]	Finnish conscripts	432	Cross-sectional	ND		5.6	(3.4-7.7)

Kataoka, 1991 Sweden [32]	Sweden	108	Cross-sectional	ND		1.9	(0-4.4)

Desai, 2011 UK [16]	General practice + GUM clinic attendees		Retrospective cohort	168		ND	

*CI *confidence interval, *GUM *Genitourinary medicine, *ND *no data

^a ^26 European countries

Other studies evaluated the prevalence of genital warts in high-risk populations [37-40]. Prevalence ranged from 2.5% in male sex partners of women with cervical intraepithelial neoplasia (CIN) in the Netherlands [37], to 58% among men who have sex with men (MSM) in Greece [38] (Table 4).

**Table 4:** Studies reporting on the prevalence of genital warts in high-risk male populations in Europe^a^

Author	Country	Population	Design	Prevalence (%)	95%CI
Kyriakis, 2005 [38]	Greece	3667HS 856 MSM 211 IDU (HS+MSM)	Cross-sectional, hospital based	53 HS 58 MSM 51 IDU	(51.4-54.6) (55.1-61.7) (44.0-57.4)

Van der Snoek, 2003 [40]	The Netherlands	258 MSM 241 HIV- 17 HIV+	Cross-sectional	27	(21.6-32.4)

Bleeker, 2002 [37]	The Netherlands	119 male sex partners of women with CIN	Prospective	2,5	(0.31-5.30)

Svare, 2002 [39]	Denmark	261 STD clinic attendees	Prospective	25	(19.7-30.1)

*HS *heterosexual, *MSM *men who have sex with men, *IDU *intravenous drug user, *CI *confidence interval, *CIN *cervical intraepithelial neoplasia, *STD *sexually transmitted disease

^a ^26 European countries

##### Incidence

Six studies [16,17,30,33,34,36] provided incidence data of new cases for men in Europe, ranging from 136 per 100,000 man-years in Spain [30] to 294 per 100,000 man-years in Sweden [36]. When considering new and recurrent cases incidence rates could even reach 319 per 100,000 man-years [33,34].

The most robust data on the incidence of genital warts come from Germany [17] and the United Kingdom [16]: Based on these studies, a lower incidence estimate of 147.6 per 100,000 man-years and an upper estimate of 167.7 per 100,000 man-years were extracted, and so new annual genital wart cases in men in Europe were estimated to range between 335,301 and 380,961. Assuming an HPV6/11 prevalence in genital warts of 85.5% [10,12], between 286,682 and 325,722 of these cases were estimated to be attributable to HPV6/11.

Recent data on genital warts rates seen at GUM clinics are available at Health Protection Agency webpage: the incidence of genital warts in men increased from 148.5 per 100,000 in 2006 to 160.5 per 100,000 in 2010 [41].

#### Recurrent respiratory papillomatosis

European data on the incidence of recurrent respiratory papillomatosis are very scarce. The only available data were reported from Denmark where two studies reported similar incidence rates: 0.38 cases per 100,000 for the period 1968-1984 [42] and 0.35 per 100,000 for the period 1974-1999 [43]. However there was no information about sex-specific incidence.

### HPV infection

#### HPV infection in the oral cavity and oropharynx

Five European studies provided data about the prevalence of oral cavity and oropharyngeal HPV infections in men. Three of them measured the prevalence of all HPV types in the oral mucosa of cancer-free men. Eike et al. [44] and Van Dornuum et al. [45] found a prevalence of 0% in the oral mucosa of almost 100 men, while in the Finnish Family Study prevalence was 18% among 131 husbands of pregnant women tested with nested PCR [46]. The two remaining studies evaluated high-risk HPV prevalence and found a 3.8% prevalence in 26 healthy adult volunteers, from whom samples had been taken at three different sites of the oral cavity [47], and 14.5% in the saliva of 69 male odonto-stomatology attendees without oral pathologies, aged 4-77 years [48]. In all studies HPV detection was carried out by PCR (Table 5).

**Table 5:** Studies reporting head and neck human papillomavirus (HPV) infection in cancer-free men in Europe^a^

Author, year, country	Sample types	Population	Sample size	Age (years)	Sampling techniques and detection methods	HPV (%)			
						**Any**	**95%CI**	**High-risk**	**95%CI**

Montaldo 2007 Italy [48]	Saliva	Odonto- stomatology attendees, non-diseased for oral pathology	69	4-77	Specimen PCR	ND		14.5	(7.17- 25.04)

Kujan 2006 UK [47]	Three different sites in the oral cavity	Healthy volunteers	26	> 18	Brushing PCR	ND		3.8	(0.10- 19.64)

Rintala 2005 Finland [46]	Oral mucosa	Husbands of pregnant women (Finnish Family Study)	131		Brushing nested PCR	18	(11.5-24.5)		

Eike 1995 Denmark [44]	Oral mucosa	Patients with unrelated diseases and their relatives	31	20-79	Smear PCR	0		0	

Van Doornum, 1992 [45]	Tongue and buccal mucosa	Men with multiple heterosexual partners	65	mean age: 38	Swabs PCR	0		0	

*ND *no data, *CI *confidence interval

^a ^26 European countries

#### Anal HPV infection

Six European studies analysed the prevalence of anal HPV infections. All included cancer-free subjects belonging to presumed high-risk groups for HPV infection, such as MSM, HIV-positive men, sexually-transmitted disease (STD) clinic attendees and heterosexual men with multiple sexual partners. The overall prevalence of anal HPV infection in these high-risk groups ranged between 15.3% in a sample of 85 male STD clinic attendees [49] and 94.1% among 17 HIV-positive MSM [49]. Anal HPV prevalence in HIV-positive MSM and HIV-positive heterosexual men ranged from 64.7% [40] to 94.1% [50] and 46,0% [51] to 68.2% [52], respectively. HPV prevalence in HIV-negative MSM was evaluated in one study to be 32.8% [40] (Table 6).

**Table 6:** Studies reporting anal human papillomavirus (HPV) infection in cancer-free men in Europe^a^

Author, year	Country	Population	Sample size	Sampling techniques and detection methods	HPV (%)	
					**Any**	**95%CI**

Van der Snoek, 2003 [40]	The Netherlands	**MSM** HIV+ and HIV-	258 (17 HIV+, 241 HIV-)	Swabs PCR + LiPA	64.7 (HIV+) 32.8 (HIV-)	(38.33-85.79) (27.06-38.94)

Pierangeli, 2008 [56]	Italy	MSM and HS (HIV+ and HIV-)	61 (36 HIV+, 25 HIV-)	Anal brushing PCR	81.6 (HIV+) 68.0 (HIV-)	(63.98-91.81) (46.50-85.05)

Sirera, 2006 [52]	Spain	**HIV+ **men (MSM and HS)	74 (52 MSM 22 HS)	Cytobrush PCR	82.6 (MSM) 68.2 (HS)	(69.67-91.77) (45.13-86.14)

Piketty, 2003 [51]	France	**HIV+ **men (MSM and HSiDU)	118 (67 MSM, 50 HSiDU)	Swabs PCR	85.1(MSM) 46.0 (HSiDU)	(74.26-92.60) (31.81-60.68)

Van Doornum, 1994 [49]	The Netherlands	STD clinic attendees	85	Cotton swabs or wooden spatula	15.3	(8.40-24.73)

Löwhagen, 1999 [50]	Sweden	STD clinic attendees (HIV+ and HIV- MSM)	30 (17 HIV+, 13 HIV-)	Cotton swabs PCR	76.7 94.1 (HIV+) 53.8 (HIV-)	(61.5-91.8) (82.9-100.0) (26.7-80.9)

*CI *confidence interval, *MSM *men who have sex with men, *STD *sexually transmitted disease, *HS *heterosexual, *HSiDU *heterosexual intravenous drug user, *STD *sexually transmitted disease

^a ^26 European countries

#### Penile HPV infection

Twenty-three European studies provided information on the prevalence of penile HPV infections in more than 4,300 men. The majority of the study subjects belonged to high-risk groups (HIV-positive men, MSM, partners of women with CIN and STD clinic attendees). The overall HPV prevalence ranged from 2.2% (95%CI: 0-6.4) in the semen of 46 husbands of women with HPV-related genital lesions [53], to 72.9% (95%CI: 65.8-79.3) in a report on male partners of women with CIN [54].

One study [55], including 947 men with HPV-related lesions or HPV-positive partners, compared samples at different anatomical sites of the penis as well as semen samples. HPV prevalence was highest on the penile shaft (58.3%), followed by the glans/corona (43.3%), urethra (31.0%) and semen (23.9%) (Table 7).

**Table 7:** Studies reporting penile human papillomavirus (HPV) infection in cancer-free men in Europe^a^

Author	Country	Anatomical site(s)	Population	Sample size	Sampling techniques and detection methods	HPV (%)			
						**Any**	**95%CI**	**High-risk**	**95%CI**

Bleeker 2005 [54]	The Netherlands	Glans, corona, frenulum and prepuce	Partners of women with CIN	181	Cervex brush GP5+/6+ PCR	72.9	(65.8-79.3)	58.5	(51.0-79.3)

Kjaer 2005 [57]	Denmark	Glans and corona	Danish soldiers	337	Penile swabs PCR	33.8	(28.8-39.2)		

Sirera 2006 [52]	Spain	Coronal sulcus, glans and urethra distal	**HIV+ **men (MSM and HS)	74 (52 MSM 22 HS)	Cytobrush PCR	38 (MSM) 32 (HS)	(25-53) (14-55)		

Van der Snoek 2003 [40]	The Netherlands	Coronal sulcus	STD clinic attendees; MSM HIV+ and HIV-	258 17HIV+ 241 HIV-	Dry swab PCR + LiPA	23.5 (HIV+) 15.8 (HIV-)	(6.81-49.9) (11.4-21.0)		

Bleeker 2002 [37]	The Netherlands	Glans, corona, frenulum and prepuce	Partners of women with CIN	119	Cervex brush GP5+/6+ PCR	59.0	(49.4-67.8)	55.4	(46.1-67.8)

Svare 2002 [39]	Denmark	Glans, corona, shaft, scrotum and perianus	STD clinic attendees	198	Scrapping plus pre-wetted swabs GP5+/6+ PCR	45.0	(37.9-52.2)	18.6	(12.5-52.2)

Wikstrom 2000 [58]	Sweden	Glans, corona, shaft, scrotum and perianus	STD clinic attendees	235	Scrapping plus pre-wetted swabs GP5+/6+ PCR	20.4	(15.3-25.6)	12.8	(8.5-17.0)

Van Doornum 1994 [49]	The Netherlands	Corona, urethra, anus and rectum	HS STD clinic attendees	85	Cotton swab/wooden spatula PCR	28.2	(19.0-39.0)		

Forslund 1999 [59]	Sweden	Urethra	Military conscripts and adolescent clinic attendees	138	Prewetted brush PCR	8.7	(4.6-14.7)		

Hippelainen 1993 [31]	Finland	Glans, prepuce, sulcus, urethral meatus and urethra	Finnish conscripts	285	Prewetted brush PCR	16.5	(12.4-21.3)		

Franceschi 2002 [60]	Spain	Glans, corona, and urethra	Husbands of women with invasive cervical cancer	84	Cotton tipped Swabs PCR	11.9	(5.9-20.8)	1.2	(0.0-20.8)

			Husbands of women with cervical carcinoma in situ	102		21.6	(14.0-30.8)	6.9	(2.8-30.8)

			Husbands of control women	168		3.6	(1.3-7.6)	1.8	(0.4-7.6)

Wikstrom 1991 [58]	Sweden	Corona, prepuce, and urethral meatus	STD clinic attendees, men with no history of genital warts	135	PCR			13.3	(8.1-59.1)

Barzon 2010 [55]	Italy	Glans/Corona, penile shaft, urethra	Males presenting for screening for STDs, investigation of suspected HPV-related lesions, or because of HPV-positive partners	947	Swabs PCR	Glans/ corona: 43.3 Penile shaft: 58.3 Urethra: 31.0 Semen: 23.9	(39.0-47,6) (47.8-68.3) (25.6-36.5) (19.0-28.8)	12.2 9.4 7.3 5.6	(9.32-15.0) (4.38-17.05) (4.2-10.4) (18.9-28.8)

Kataoka, 1991 [32]	Sweden	Urethra	Army conscripts	105	Brushing PCR	17.1	(9.9-24.4)		

Hillman, 1993 [61]	United Kingdom	Urethra Urine	STD clinic attendees	100 urethra samples 88 urine specimen	Cotton-tipped swabs Urine sample PCR	Urethra: 18.0 Urine: 12.5	(10.5-25.5) (5.6-19.4)	Urethra: 12.0 Urine: 9.1	(5.6-18.4) (3.1-15.1)

Aynaud, 2002 [53]	France	Semen	Men with normal peniscopy whose female partners have genital HPV lesions	46	Ejaculate PCR	2.2	(0-6.4)	NI	

Aynaud, 2003 [62]	France	Meatal-urethral smears	Men with normal peniscopy whose female partners have genital HPV lesions	34	Brushing PCR	2.9	(0-8.4)	NI	

Giovanelli, 2007 [63]	Italy	Penile shaft, foreskin, coronal sulcus, frenulum, glans, semen	Partners of HPV-positive women	50	Cotton-tipped swab, cytobrush, ejaculate PCR	72.0	(59.6-84.4)	56.0	(42.2-69.8)

Benevolo, 2008 [64]	Italy	Penile shaft, prepuce, coronal sulcus, glans, distal urethra	Male partners of women with CIN and/or positive HPV	71	Cytobrush PCR	35.2	(24.1-46.3)	31.0	(20.2-41.7)

Bleeker, 2004 [65]	The Netherlands	Glans, corona, sulcus, frenulum, foreskin	Men with female partners, visiting department of dermatology for non-STD complaints	83	Brushing PCR	25.3	(15.9-34.7)	19.3	(10.8-27.8)

Castellsagué, 1997 [66] Bosch, 1996 [67]	Spain	Intrameatal and distal urethra, glans, coronal sulcus	Male partners of women with cervical cancer	183	Cotton-tipped swabs	17.5	(12.0-23.0)	15.8	(10.6-21.1)

			Male partners of control women	171		3.5	(0.8-6.3)	2.3	(0.1-4.6)

Voog, 1997 [68]	Sweden	Glans penis, prepuce	STD clinic attendees	20	Cytobrush	25	(6.0-44.0)	NI	

Strand, 1993 [69]	Sweden	Urethra, glans penis, sulcus, preputium, penile shaft	STD clinic attendees	65	Plastic probe + cytobrush	29.2	(18.2-40.3)	NI	

*CI *confidence interval, *CIN *cervical intraepithelial neoplasia, *MSM *men who have sex with men, *HS *heterosexual, *STD *sexually transmitted disease

^a ^26 European countries

## Discussion

Using different data sources and extrapolation methods, this review showed that the overall estimated epidemiological burden of HPV-related cancers and non-malignant diseases is high among men in Europe. In addition to malignant diseases, which include a subset of head and neck cancers and anal and penile cancers, non-malignant diseases such as genital warts and recurrent respiratory papillomatosis are also associated with HPV. Moreover, the majority of HPV-positive cancers in men are attributable to oncogenic HPV16/18, while the great majority of genital warts and virtually all cases of recurrent respiratory papillomatosis are caused by HPV6/11.

In this report, a total of 72,694 new HPV-related cancer cases were estimated to occur each year in men in Europe. Moreover, we estimated that 17,403 of these cancers could be attributable to HPV, of which 15,497 were estimated to be specifically attributable to HPV16/18.

In addition, between 335,301 and 380,961 new cases of genital warts were estimated to occur annually in men in Europe, with 286,682-325,722 of them attributable to HPV6/11.

Recurrent respiratory papillomatosis is a very rare disease and incidence data in Europe are scarce. It was not possible to estimate the number of new annual cases in men in Europe. Nevertheless, the association with HPV is particularly strong for this disease, and virtually all cases are attributable to HPV6/11 [70].

### HPV-related cancers

#### Head and neck cancers

There was high variability in the incidence of head and neck cancers, irrespective of HPV status, with age-standardised incidence rates in Europe ranging from 5.6 to 33.0 per 100,000 man-years. The distribution of the subset of head and neck cancers included herein also varied throughout Europe. This may be partially due to regional differences in HPV prevalence, which are in turn related to differences in the distribution of risk factors for HPV infection, such as sexual behaviour. It may also be due to differences in the distribution of non-HPV-related risk factors like alcohol consumption and tobacco use, which explain some 80% of these cancers.

Large cohort studies from Sweden show increasing evidence of a steady rise in the incidence of a subset of cancers of the oral cavity (especially lateral border of the tongue) [71] and oropharynx (notably tonsillar and base of the tongue [22,23,71,72]). In these studies oropharyngeal cancer was most strongly associated with HPV. The overall HPV detection rate in tonsillar carcinoma reached up to 51%, with HPV16 being the most common type (84%) [71,73,74]. The increasing trend may be due to an increase of HPV infections in the head and neck, possibly related to changes in sexual behaviour.

#### Anal cancer

An overall number of 2,162 annual cases were estimated to occur in men in Europe, irrespective of HPV status and the association of anal cancer with HPV is extremely strong (84.2% HPV-related) [19].

An increasing trend in the incidence of anal cancer has been reported for a number of countries, including Denmark [25], Sweden [27], the Netherlands [28] and the United Kingdom [24]. Regional differences in incidence could be explained by regional differences in anal HPV prevalence, the key causal precursor for anal cancer [75], and its associated causes and consequences, such as history of condyloma [76], history of anoreceptive intercourse [76,77] and increased lifetime number of sexual partners [78].

#### Penile cancer

Three thousand one hundred seventy-eight new penile cancer cases, irrespective of HPV status, were estimated to occur yearly in Europe with almost half of these cases (1,484) attributable to HPV.

In addition to regional differences in penile HPV prevalence, differences in the incidence of penile cancer may be due to differences in the regional distribution of risk factors that are not related to HPV infection, such as smoking, phimosis and absence of circumcision [79,80].

### HPV-related non-malignant diseases

#### Genital warts

The burden of genital warts in men in Europe is substantial. Extrapolated from the most robust European incidence data collected in Germany and in the United Kingdom, new cases were estimated to range between 335,301 and 380,961 in men in Europe every year. The vast majority are estimated to be attributable to HPV6/11. As for HPV-related malignant diseases, the incidence of genital warts varies highly by geographical region. This may be due partially due to differences in the distribution of risk factors for HPV infection. Furthermore, there is some evidence of a steady rise of genital warts in men in the United Kingdom [81].

Genital warts are not life-threatening, but they are associated with high morbidity: psychosocial stigma, psychosexual dysfunction, depression and lower quality of life [82,83]. Treatment is painful, and there is a high risk of recurrence.

#### Recurrent respiratory papillomatosis

In Europe, only two studies provided data on the incidence of recurrent respiratory papillomatosis in both sexes: 0.35-0.38 per 100,000 person-years. There was no specific information about incidence in men but the sex ratio for juvenile onset recurrent respiratory papillomatosis was reported to be 1:1, while there was a pronounced male preponderance in onset in adulthood [42].

Virtually 100% of cases are caused by HPV, the most common types being HPV6/11 [11]. Maternal condyloma or genital HPV infection during pregnancy is the overwhelming risk factor for juvenile onset recurrent respiratory papillomatosis (more than 200-fold increased risk) [43,70]. Although non-malignant, the disease is associated with very high morbidity. It has the potential to be life-threatening and the number of lifetime surgeries may exceed 100 in children with severe disease [70].

### HPV infection

#### Head and neck

Overall HPV prevalence in the head and neck ranged from 0 to 18% among men in Europe, and specific prevalence of all high-risk HPV types from 0 to 14.5%. Due to the small number of European studies and the relatively small sample sizes, these results must be considered with caution. More robust data are available when worldwide studies are considered. Indeed, a systematic review of all published studies worldwide (n = 18) that detected HPV DNA in the oral cavity of 4,070 cancer-free subjects found a pooled HPV prevalence (any type) of 4.5% (95%CI: 3.9%-5.1%); the prevalence of high-risk types was 3.5% (95%CI: 3.0%-4.1%) [84]. In a recent study of 1,680 healthy men from the United States, Mexico, and Brazil, HPV DNA was detected in the oral cavity of 4.0% (95%CI: 3.1%-5.0%), and carcinogenic HPV DNA in 1.3% (95%CI: 0.8%-2.0%) of subjects [85].

#### Anus

The prevalence of anal HPV infection in men in European studies ranged between 15.3% [49] and 94.1% [50], but all these studies included presumably high-risk populations and there were no data on the prevalence of anal HPV infection in the general male population in Europe.

#### Penis

The prevalence of penile HPV infection in men in Europe ranged from 2.2% (95%CI: 0.0%-6.4%) [53] to 72.9% (95%CI: 65.8%-79.3%) [54]. The heterogeneity of the published data is partly due to the use of different sample techniques, detection methods, anatomical sites or specimens sampled, as well as study populations with different risk factors. The penis is made up of different types of body tissue, which differ in their susceptibility to HPV infection. Data from literature have indicated that the prepuce has the highest proportion of HPV-positive samples, though reports have also shown an increase in HPV DNA detection when multiple anatomical sites were sampled [86].

Most of the studies that evaluated the prevalence of HPV infection in anogenital sites included high-risk populations. Nevertheless, data from the United States have shown a very high prevalence of anogenital HPV infection (65%) in population-based studies [87,88]. The incidence of new genital HPV infections in this population was 38.4 per 1,000 person months (95%CI: 34.3-43.0) in a recent study including men aged 18-70 years with a median duration of HPV infection of 7.52 months (95%CI: 6.80-8.61) for any HPV infection and 12.19 months (95%CI: 7.16-18.17) for infection with HPV16 [89].

In addition, a recent global review of the age-specific prevalence of HPV infection in men [86] identified 64 abstracts with data on genital HPV infection in men worldwide, including 38 from populations at high risk of HPV infection. The authors of the study concluded that the risk of HPV infection is generally high for most men in many geographical areas, with comparable prevalence in both low-risk (1-84%) and high-risk populations (2-93%).

Little is known about the natural history of HPV infection in men. Few studies have prospectively assessed HPV infection, and therefore few data are available on the incidence, acquisition and persistence of HPV infection in men. A recent review on HPV prevalence in men concluded that, in contrast to results for women, age-specific prevalence curves remained relatively flat with age, or declined only slightly with post-peak prevalence. Thus, men may potentially have more long-term persistent HPV infections, or a higher rate of re-infection [86].

### Comparison to the burden in women

For comparison purposes, the methods described above were also used to estimate the epidemiological burden of HPV-related cancers and genital warts in women in Europe. Using our methods of estimation, a total of 32,562 cancer cases specifically due to HPV16/18 were expected to occur in women in Europe every year. The new annual number of cases of genital warts attributable to HPV6/11 in women in Europe was estimated to range between 288,959 and 388,873.

A total of 48,059 HPV16/18-positive cancer cases were estimated to occur annually in Europe in both sexes, of which more than 30% occur in men (Table 8). Other than cervical cancer, 23,254 annual cases of which are estimated to be HPV16/18-positive, the estimated burden of cancer cases attributable to HPV is higher in men than in women, and is mainly driven by head and neck cancers.

**Table 8:** The burden of new yearly human papillomavirus (HPV)16/18-related cancers in men vs. women in Europe^a^

Anatomical site (ICD-10 code)	Men Number of new yearly cases (% of overall burden in both sexes)	Women Number of new yearly cases (% of overall burden in both sexes)
**Cervix uteri **(C53)	0 **(0)**	23,254 **(48.4)**
**Head and neck **(C01-C02, C03-C06, C09, C10, C12, C13, C14 and C32)	12,706 **(26.4)**	2,531 **(5.3)**
**Anus **(C21)	1,700 **(3.5)**	2,929 **(6.1)**
**Vulva **(C51)	0 **(0)**	2,702 **(5.6)**
**Vagina **(C52)	0 **(0)**	1,146 **(2.4)**
**Penis **(C60)	1091 **(2.3)**	0 **(0)**

**Total**	15,497 **(32.2)**	32,562 **(67.8)**

*ICD *International Classification of Diseases

^a ^26 European countries

The incidence of head and neck cancers attributable to HPV16/18 is five-fold higher in men (12,707 new cases yearly) than women (2,531 new cases yearly). In addition, new cases of genital warts attributable to HPV6/11 in both genders were estimated to range between 614,681 and 675,555 yearly in Europe, half of them affecting men.

### Impact of HPV vaccination

The preliminary effects of HPV vaccination on genital warts and precancerous lesions have been reported from Australia, where a vaccination programme using the quadrivalent HPV vaccine was implemented. The data showed that in addition to a significant decline in the number of cases of genital warts and in the incidence of high-grade cervical abnormalities among young women in Australia, the number of cases of genital warts among heterosexual men also declined markedly [90].

### Limitations

Our report has several limitations. A short-term prediction method was used to estimate the expected number of incident cancer cases in 2008 from the most recent data collected from 1998 to 2002. Therefore these predictions were accurate only if the disease rates remained stable over time. In the case of an increasing trend, they would slightly underestimate the expected number of cases, and the opposite would be true in the case of a decreasing trend.

As mentioned above, the CI5 database contains national cancer incidence rates for 17 European countries. For the remaining nine countries included in this report only regional incidence rates were available in the CI5 database. Although we assessed the geographical coverage and distribution of these regional registries, other non-controllable factors, like differences in alcohol and tobacco consumption, could vary and influence regional incidence rates. The results should thus be interpreted with particular caution.

The presence of HPV DNA is used to calculate the prevalence of HPV in a given population. However the mere presence of HPV is insufficient to prove causation, as the infection may be transient and not related to the carcinogenic process. Therefore our application of previously published HPV prevalence to an estimated number of new cancer cases may have yielded an overestimation of cases attributable to HPV infection. Finally, the prevalence of HPV16/18 co-infections has not been evaluated, thus summarising HPV16/18 prevalence may have led to an overestimation of the cancer burden attributable to these types.

Another limitation is the absence of sex-specific HPV prevalence data in anal and head and neck cancers. Consequently, our report assumed that HPV prevalence is the same in both sexes. Currently, we have no reliable data that would confirm this hypothesis, and it seems to be inconsistent with the worldwide data from the meta-analysis by De Vuyst et al. [19], which showed a higher HPV prevalence in anal carcinoma among women (90.8%) than men (74.9%). To our knowledge, sex-specific data on HPV prevalence in head and neck cancers are lacking, and future research is needed.

We applied the same HPV prevalence to all European countries in this report. However, regional differences in HPV prevalence could exist, notably due to the fact that in some countries other non-HPV-related risk factors, i.e., tobacco or alcohol consumption may predominate. Furthermore, when type-specific HPV data for Europe was lacking, we used worldwide data, thus assuming that there is no difference in the HPV16/18 distribution between Europe and other parts of the world.

The method used to estimate the expected annual number of new genital wart cases attributable to HPV6/11 has also some limitations. We based our estimations on incidence data extracted from only two studies and only one of them was population-based [17]. In addition, due to the lack of data, it was not possible to extrapolate incidence data per age, but only by sex. However, there may be important differences in the age structure of European countries. Also, the incidence of genital warts throughout Europe may vary, due to regional differences in the prevalence of HPV infection and its underlying risk factors.

## Conclusions

A total of 15,497 new cancer cases attributable to HPV16/18, and between 286,682 and 325,722 new cases of genital warts attributable to HPV6/11, were estimated to occur yearly in men in Europe.

The burden of HPV-related cancer in men is higher than generally perceived and is primarily driven by head and neck cancers. There is currently no routine screening for HPV-related cancers in men, and head and neck cancers in particular are associated with very high morbidity. In addition, there is some evidence of a steady rise of the incidence of HPV-related cancers and genital warts.

The high proportion of HPV-positive cancers attributable to HPV16/18, and of non-malignant diseases attributable to HPV6/11, underscores the potential to prevent the majority of HPV-related diseases among men through prophylactic vaccination with the quadrivalent HPV vaccine. Further research is needed to evaluate the efficacy of prophylactic HPV vaccines at non-cervical sites in men and women.

## Abbreviations

CI: Confidence interval; CI5: Cancer Incidence in Five Continents; CIN: Cervical intraepithelial neoplasia; GUM: Genitourinary medicine clinics; HPV: Human papillomavirus; IARC: International Agency for Research on Cancer; MSM: Men who have sex with men; STD: Sexually transmitted disease.

## Competing interests

SH and GDF are employed by Sanofi Pasteur MSD. SS has consulted Sanofi Pasteur MSD with regard to HPV in human diseases. MB has received occasional transport to attend scientific meetings from Glaxo Smith Kline. XC has received travel and speaker honoraria and investigator grants through ICO from Merck & Co. Inc., Glaxo Smith Kline and Sanofi Pasteur MSD.

## Authors' contributions

SH contributed to the study design, literature research, data-analysis, interpretation of findings and drafting of the manuscript. SS contributed to the study design, interpretation of findings and critical editing of the manuscript. GDF contributed to the study design, interpretation of findings and critical editing of the manuscript. MB contributed to the data-analysis, interpretation of findings and critical editing of the manuscript. XC contributed to the study design, data-analysis, interpretation of findings and critical editing of the manuscript. All authors critically reviewed the manuscript and approved the final version.

## Pre-publication history

The pre-publication history for this paper can be accessed here:

http://www.biomedcentral.com/1471-2407/12/30/prepub
